# Intravenous immunoglobulin treatment stabilizing a patient with Anti-PL7 antisynthetase syndrome with interstitial lung disease and eosinophilic inflammation

**DOI:** 10.1016/j.rmcr.2022.101686

**Published:** 2022-06-14

**Authors:** Soran Peshbahar, Charlotte Hyldgaard, Elisabeth Bendstrup

**Affiliations:** a Diagnostic Centre, Silkeborg Regional Hospital, Falkevej 1-3, 8600, Silkeborg, Denmark; b Center for Rare Lung Diseases, Department of Respiratory Diseases and Allergy, Aarhus University Hospital, Palle Juul-Jensens Boulevard 99, 8200, Aarhus N, Denmark

**Keywords:** Antisynthetase syndrome (AS), Interstitial lung disease (ILD), Intravenous immunoglobulin (IVIG), Anti-PL7, Eosinophilic inflammation

## Abstract

Antisynthetase syndrome (AS) is a rare autoimmune disease characterized by autoantibodies against aminoacyl-transfer RNA synthetase and clinical features which can include interstitial lung disease (ILD). Current available evidence of treatment is based on expert opinions and case reports. Here, we present a patient with an initial diagnosis of eosinophilic pneumonia, who was later diagnosed with anti-PL7 antisynthetase syndrome with ILD and eosinophilic inflammation. The patient was non-responsive to classic immunosuppressants but responded remarkably well to intravenous immunoglobulin.

## 1 Introduction

Antisynthetase syndrome (AS) is a rare autoimmune disease characterized by autoantibodies against aminoacyl-transfer RNA synthetase and clinical features which can include inflammatory myositis, interstitial lung disease (ILD), Raynaud's phenomenon and arthritis [1]. The prevalence of antisynthetase syndrome is difficult to determine as the disease only recently have been studied as a separate entity among autoimmune inflammatory myopathies. ILD in patients with AS is often more severe compared to polymyositis- and dermatomyositis-associated ILD [2]. The available evidence of treatment is based on expert opinions and case reports [1]. Here, we present a patient who was initially treated on suspicion of eosinophilic pneumonia, but was later diagnosed with anti-PL7 antisynthetase syndrome with ILD and eosinophilic inflammation. The patient was non-responsive to classic immunosuppressive agents, but responded remarkably well to intravenous immunoglobulin (IVIG). To our knowledge, this is the first description of AS-ILD with eosinophilic inflammation in the lung.

## 2 Case report

A 55-year-old female was hospitalized in early 2015, presenting with increasing shortness of breath, fatigue and proximal muscle pain during the past 6 months. She had a family history of asthma, and no past medical history, except from allergy to house dust mite. There were no relevant exposures.

At admission, vital signs showed a temperature of 37.7 °C, saturation 93% on room air, respiratory rate 20 breaths per minute, blood pressure 119/64 mmHg and pulse 93 beats per minute.

Physical examination showed bilateral basal crackles on auscultation and clubbing of the fingers. Skin and joint examination was normal.

Pulmonary function test (PFT) showed restriction and reduced diffusion capacity. Initial six-minute walk test (6MWT) showed a reduced distance and significant desaturation (Table 1).

**Table 1:** Baseline pulmonary function and 6-min walk test.

FEV1	1.31 L (57% predicted)
FVC	1.58 L (58% predicted)
TLC	2.6 L (56% predicted)
DLCO	41% predicted
6MWTD distance	335 m
6MWT saturation	89-81%

FEV1: Forced Expiratory Volume in 1 second; FVC: Forced Vital Capacity; TLC: Total Lung Capacity, DLCO: Diffusing capacity for carbon monoxide; 6MWD: 6 minute walk distance; 6MWT saturation highest saturation and lowest saturation.

Initial blood tests including broad serology showed slightly elevated inflammatory parameters with eosinophilia, slightly positive antinuclear antibody (ANA) and elevated creatine kinase. Saturation on arterial blood gas with 2 L’ continuous oxygen therapy was 98% (Table 2). Poly-dermatomyositis blood samples with 15 specific autoantibodies were all negative. Allergy blood tests for common inhalation allergens were only positive for house dust mites.

**Table 2:** Initial blood tests.

Analysis	Result	Reference range
C-reactive protein	13.1 mg/L	<8 mg/L
Leucocytes	14.3 × 10^9^/L	3,5–10 × 10^9^/L
Eosinophils	1.22 × 10^9^/L	<0.5 × 10^9^/L
Creatinine	57 μmol/L	60–105 μmol/L
Albumin	27 g/L	36–45 g/L
Alanine aminotransferase	41 U/L	10–70 U/L
Lactate dehydrogenase	235 U/L	105–205 U/L
Creatine Kinase	305 U/L	50–200 U/L
IgE	263 × 10^3^ int.u/L	<115
ANA	1.2 (Ratio)	<1.0
O2-saturation	98%	92–99%

O_2_-sat: saturation on arterial blood gas (with 2 L continuous oxygen therapy).

**Fig. 1:**
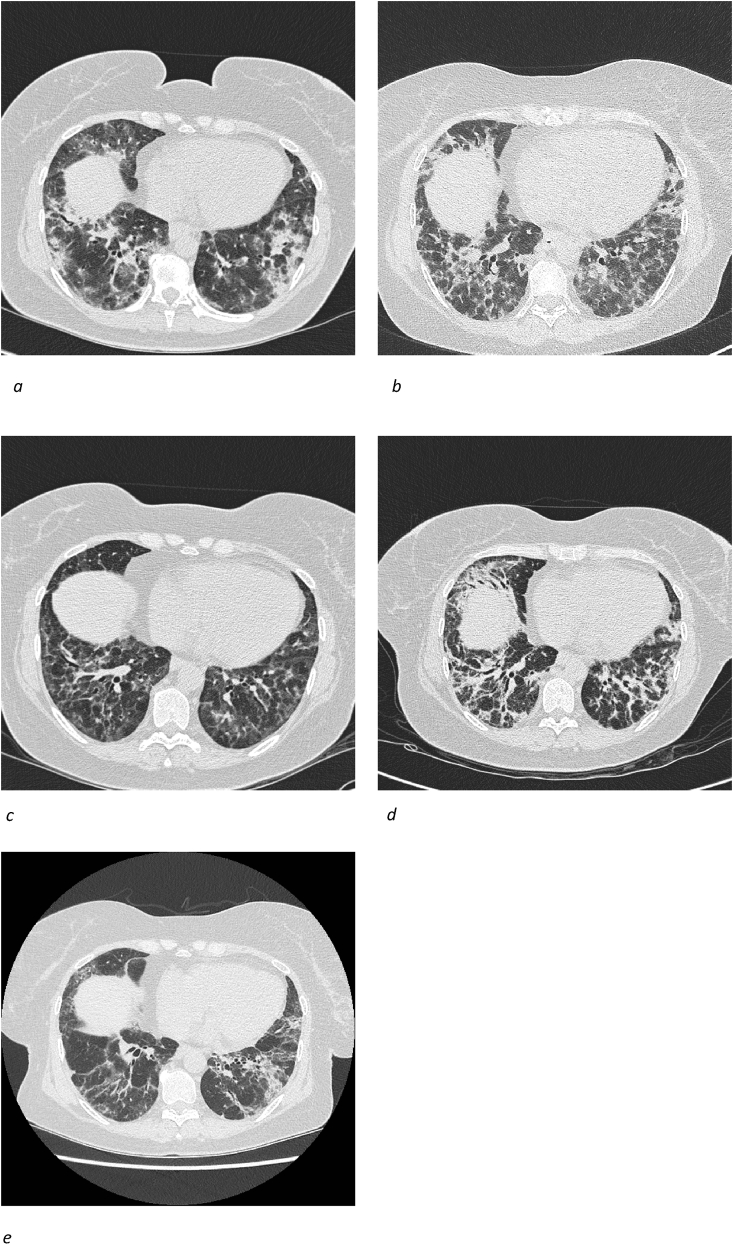
High resolution computed tomography (HRCT) of the lungs with interstitial changes during the treatment. Fig. 1a: *HRCT with interstitial lung disease with bibasilar predominance of peribronchial consolidations suspect of organizing pneumonia (OP) and non-specific interstitial pneumonia (NSIP);*Fig. 1b*: CT scan with progression of the interstitial findings;*Fig. 1c*: Significant regression of the interstitial changes after methylprednisolone;*Fig. 1d*: Basal reticulation, ground-glass opacities and perilobular consolidations consistent with OP and NSIP;*Fig. 1e*: HRCT with significant regression of the interstitial findings.*

A high resolution computed tomography (HRCT) scan was consistent with organizing pneumonia (OP) and nonspecific interstitial pneumonitis (NSIP) (Fig. 1a).

Transbronchial lung biopsy showed inflammation with eosinophilia, and cytology from bronchoalveolar lavage (BAL) showed pronounced eosinophilia with 79% eosinophils.

No infectious agents were found in culture or polymerase chain reaction analysis of BAL or blood samples.

The patient was initially treated with oral corticosteroids (OCS), prednisolone 37.5 mg/day on suspicion of chronic eosinophilic pneumonia with improvement of the respiratory symptoms and full remission of muscle symptoms. The patient was discharged after 6 days. However, the respiratory symptoms worsened during OCS tapering and she was hospitalized again. A CT angiography showed progression of the interstitial changes without signs of pulmonary embolism (Fig. 1b).

The OCS dose was increased without significant effect. DLCO had declined to 29% predicted, leading to initiation of methylprednisolone pulse therapy (MPT) of 500 mg for three days every second week (Table 3). Moreover, treatment with weekly methotrexate (MTX) as a steroid sparing agent was initiated.

**Table 3:**
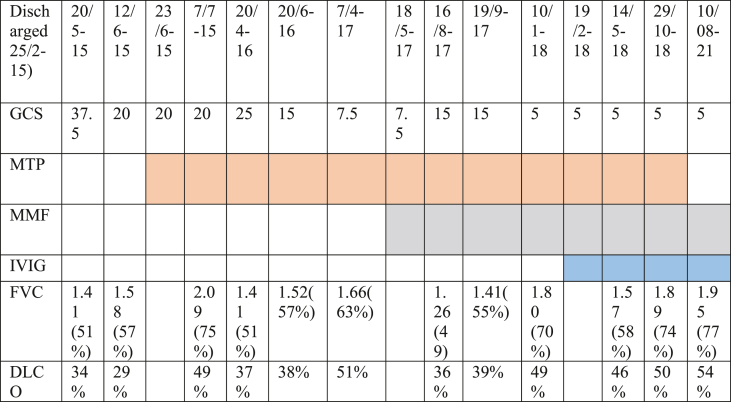
The table shows some of the visits at the outpatient clinic with lung function tests. Colored cells shows date of initiation and duration of treatment. GCS: Glucocorticosteroid, daily dose of prednisolone in mg after the patient was discharged; MTP: Methylprednisolone pulse therapy of 500 mg for three days; IVIG: Intravenous immunoglobulin at a dose of 30 g; FVC: Forced Vital Capacity; DLCO: Diffusing capacity for carbon monoxide. Methotrexate, tacrolimus and rituximab are not shown in this table.

The symptoms improved after the first course of pulse therapy with normalization of the inflammatory parameters and significant regression in the interstitial features on HRCT (Fig. 1c). After six months with 11 MPT courses, OCS was gradually tapered from 20 to 2.5 mg/day.

From February to April 2016, the patient's respiratory symptoms and lung function worsened as the interval between MPT treatments increased from 4 weeks to 8 weeks.

The daily OCS dose was increased, and a bronchoscopy with cryobiopsy and BAL was performed. The BAL cytology showed mixed cellularity with 35% eosinophils and 22% lymphocytes. Histology showed chronic inflammation and eosinophilia.

Repeat rheumatologist evaluation did not identify abnormal extrapulmonary findings, despite the initial muscle symptoms and elevated muscle enzymes. However, blood samples showed anti-SSA/Ro > 240 kU/l, ANA ratio 4.5 and on repeated myositis antibody screening, anti-PL7 was highly positive.

A new HRCT (Fig. 1d) was consistent with NSIP, and based on symptoms, clinical findings, autoimmune serology and HRCT, a diagnosis of PL7-Antisynthetase syndrome with OP/NSIP was made.

The treatment was intensified with the addition of tacrolimus (TAC) instead of MTX, and MPT was now given every four weeks for eight months with improvement of symptoms and pulmonary function. Again, the symptoms worsened after discontinuation of the MTP courses, and at this point, a combination of rituximab and mycophenolate mofetil (MMF) instead of TAC was initiated. Unfortunately, it was without improvement of symptoms, and a new HRCT confirmed progression. MPT, which until now had been the most effective treatment, was initiated again with significant effect.

Finally, intravenous immunoglobulin (IVIG) at a dose of 30 g was initiated every 3–4 months in combination with MMF 1500 mg twice daily resulting in improvement of symptoms, lung function and HRCT (Fig. 1e), and the MTP courses were discontinued.

The past three years, the patient has been stable and IVIG is now given every four months.

## 3 Discussion

No prospective randomized controlled trials have been performed in patients with AS-ILD and management is based on case series and single case reports and treatment recommendations for AS are usually extrapolated from DM and PM. Glucocorticoids are regarded as first line treatment, but when used as monotherapy in AS, relapses are frequent upon tapering, as seen in the present case. Cyclophosphamide, mycophenolate mofetil, azathioprine, tacrolimus and rituximab are frequently used steroid-sparing agents [1]. IVIG, which is IgG molecules produced from pooled plasma from a large number of healthy blood donors [3], has shown to be an effective treatment in several autoimmune inflammatory disorders [[4], [5], [6]]. IVIG exerts anti-inflammatory and immunomodulatory effects through multiple mechanisms, including inhibition of pathogenic autoantibodies, inflammatory cytokines and complement activation [7]. Furthermore, IVIG does not increase the risk of opportunistic infections.

Previously published case reports have shown beneficial effects of IVIG. A patient with severe respiratory failure due to Anti-Jo1 AS-ILD who was treated successfully with intravenous immunoglobulin, recovered from the acute phase and remained stable for years [8] (Table 4).

**Table 4:** Case reports on AS-ILD treated with IVIG. a: the dose is not further specified in the case report, CYC: cyclophosphamide, MTX: methotrexate, CyA: Cyclosporine A. AZA: Azathioprine, CS: Corticosteroid (do not include pulse therapies), MPT: Methylprednisolone Pulse Therapy.

Study	Type	Disease	Number of patients	IVIG dosage	Co-medications	Outcome
Peshbahar et al. [8]	Case report	Anti-Jo-1 AS-ILD	1	30g, 12 week intervals	CS and AZA	Recovering acute phase, survived >4 years
Huapaya et al. [9]	Retrospective study	Anti Jo-1, PL-7, PL-12 and EJ AS-ILD.	17	400 mg/kg/day for 5 consecutive days pr. Month for 6 months.	CS, AZA, MTX, MMF	Increase FVC, DLCO and decrease mean GCS dose over time
Hervier et al. [13]	Case report	Anti-Jo1 AS-ILD	1	200g x 2^a^	CYC and MTX	No effect
Riveiro-Barciela et al. [10]	Case report	Anti-Jo-1 AS-ILD	1	Unspecified dose	Cyclosporine A, CS	10% increase in FVC
Spath et al. [11]	Case reports	Anti-Jo-1 AS-ILD	3	1–5x30–40 g up to 9 courses	CS, CYC	Improvement of dyspnea and chest CT (in 2 patients)
Takai et al. [12]	Case report	Anti-Jo-1 AS-ILD	1	20 g for 5 days	CS, MPT, CYC, CYa, Polymyxin B	Recovered acute phase, improvement of dyspnea and interstitial changes
Suzuki et al. [14]	Case report	Anti-Jo-1 AS-ILD.	1	0.4 g/kg/day	CS, MPT, CYa	Temporary response with increasing PaO2/FiO2. Died 47 days after onset

Huapaya et al. [9] presented a retrospective study of 17 patients with progressive AS-ILD, refractory to other immunosuppressive drugs and treated with protocolized IVIG infusions. IVIG increased FVC, DLCO and decreased the mean prednisolone dose over time. Three other AS-ILD case reports [[10], [11], [12]] describe clinical improvement after treatment with IVIG. However, Hervier et al. [13] reported a case of Anti-Jo1 AS-ILD with no response to IVIG who responded to mycophenolate.

Suzuki et al. [14] presented a case with severe AS-ILD, where IVIG was used as salvage therapy for refractory disease. The patient had a transient increase in PaO2/FiO2 ratio but died 47 days after the disease onset. The patient had lower vital capacity and PaO2/FiO2 ratio at the time of diagnosis, compared to the survivors with PM-DM associated-ILD presented in the report.

In the present case, the patient had eosinophilia, positive anti-PL7 and severe ILD, and responded remarkably well to IVIG treatment. To our knowledge, no published case reports exist with anti-PL7 AS-ILD and eosinophilic lung inflammation. Saito et al. reported a case of anti-PL7 AS with eosinophilic pleural effusion treated with MPT, cyclophosphamide and MMF with remission [15].

Initially, the patient in the present case had a working diagnosis of eosinophilic pneumonia based on the high BAL eosinophilic count and no signs of CTD. It was discussed whether her dust mite hypersensitivity could explain the eosinophilic inflammation. However, the suspicion of CTD-associated ILD was maintained due to the patient's gender and age, and after 18 months, she was diagnosed with anti-PL7 AS-ILD. This case highlights the differential diagnostic difficulties between subgroups of ILD, and the need for continued focus and suspicion in order to reach the correct diagnosis. Anti-PL7 is a less commonly identified antisynthetase antibody, which is associated with severe ILD and treatment failure to standard immunosuppressive therapy [1]. Moreover, anti-SSA/Ro was highly positive, which is associated with AS with severe ILD [16]. In previous publications (Table 4), IVIG was administered in different doses and in combination with other immunosuppressive agents. There are no prospective trials and no reports of IVIG as first line treatment in AS-ILD patients. The lack of randomized controlled trials and the different treatment approaches in previous reports makes it difficult to choose the best treatment combination, and further studies are needed.

Anti-PL7 antisynthetase syndrome associated ILD is rare, especially with eosinophilic inflammation. The present case indicates that IVIG can be a treatment option.

## Funding

This research did not receive any specific grant from funding agencies in the public, commercial, or not-for-profit sectors.

## Declaration of competing interest

None.
